# Pregnancy outcomes before and during COVID-19 pandemic in Tamale Metropolis, Ghana: A retrospective cohort study

**DOI:** 10.1371/journal.pone.0302589

**Published:** 2024-04-30

**Authors:** Obed Duah Kwaku Asumadu, Michael Boah, Dennis Chirawurah, Joyce Aputere Ndago, Vida Nyagre Yakong, David Abatanie Kanligi, Martin Nyaaba Adokiya

**Affiliations:** 1 Department of Social and Behavioural Change, School of Public Health, University for Development Studies, Tamale, Ghana; 2 Department of Epidemiology, Biostatistics and Disease Control, School of Public Health, University for Development Studies, Tamale, Ghana; 3 Department of Occupational and Environmental Health, School of Public Health, University for Development Studies, Tamale, Ghana; 4 Department of Preventive Health Nursing, School of Nursing and Midwifery, University for Development Studies, Tamale, Ghana; 5 Pediatric Unit, Savelugu Municipal Hospital, Ghana Health Service, Tamale, Northern Region, Ghana; University of Cape Town, SOUTH AFRICA

## Abstract

**Background:**

The COVID-19 pandemic affected expectant mothers seeking maternal health services in most developing countries. Access and utilization of maternal health services including antenatal care (ANC) attendance and skilled delivery declined drastically resulting in adverse pregnancy outcomes. This study assessed pregnancy outcomes before and during COVID-19 pandemic in Tamale Metropolis, Ghana.

**Methods/Design:**

A retrospective cohort study design was employed. A random sampling technique was used to select 450 women who delivered before or during the COVID-19 pandemic in Tamale Metropolis, Ghana. The respondents were interviewed using structured questionnaire at their homes. In this study, the data collected were socio-demographics characteristics, ANC attendance, before or during pandemic delivery, place of delivery and birth outcomes. Chi-square test and bivariate logistic regression analyses were performed under significant level of 0.05 to determine factors associated with the outcome variables.

**Result:**

Of the 450 respondents, 51.8% were between 26 and 30 years of age. More than half (52.2%) of the respondents had no formal education and 93.3% were married. The majority (60.4%) of the respondents described their residence as urban setting. About 31.6% of the women delivered before the pandemic. The COVID-19 pandemic influenced place of delivery. The proportion of women who attended at least one ANC visit (84.5% before vs 70.5% during), and delivered at a hospital (76.8% before vs 72.4% during) were higher before the pandemic. More women were likely to deliver at home during COVID-19 (OR: 2.38, 95%CI: 1.52–3.74, p<0.001). Similarly, there was statistically significance difference between before and during COVID-19 delivery on at least one ANC attendance (OR: 2.72, 95%CI: 1.58–1.67, p<0.001). Women who delivered during COVID-19 were about twice more likely to develop complications (OR: 1.72, 95%CI: 1.03–2.87, p = 0.04).

**Conclusion:**

ANC attendance and health facility delivery decreased while pregnancy complications increased during COVID-19. During disease outbreaks, outreach engagement strategies should be devised to increase access and utilization of maternal health services for marginalized and underserved populations. The capacity of health workers should be strengthened through skills training to manage adverse birth outcomes.

## Introduction

The severe acute respiratory syndrome coronavirus 2 (SARS-CoV-2) caused Coronavirus Disease 2019 (COVID-19) which continues to be a major global health issue [1, 2]. Everyone was at risk of infection and developing serious complications from the condition. This necessitated the World Health Organization (WHO) to declare the condition a public health emergency. As of May 9, 2020, the virus had infected 57,870 Africans, resulting in 2,154 fatalities and 19,363 recoveries [3]. Ghana was the first and fifth most afflicted country in West Africa and the whole of Africa respectively [4, 5]. As a result, the Government of Ghana implemented interventions in accordance with WHO’s recommendations such as closing of all borders. Additionally, community enhanced surveillance, mandatory quarantining of all international travelers and mandatory self-quarantine for those who had come into close contact with a sick person or suspected case were conducted in Ghana [6]. Reports from WHO indicated that many countries experienced partial disruptions in prenatal care and skilled delivery services during the outbreak of COVID-19 [7]. Many health facilities may have experienced a decline in antenatal care (ANC) attendance due to lockdowns, financial difficulties and fear of SARS-CoV-2 infection. In Ghana, healthcare delivery was disrupted in many domains, especially maternal health services during this period [8]. This was a major source of concern for the public and healthcare professionals.

The impact of the pandemic on ANC attendance was enormous and have been explored in a previous study [9]. These issues revolved around individual factors (such as a decreased ability to pay for care), facility-level factors including shutdowns, lack of healthcare providers, or prerequisites for entry (wearing masks and getting tested), and policy-level factors (mobility restrictions) [9]. For the purpose of creating effective interventions to promote care-seeking, it is essential to determine how these complicated aspects influenced women’s decisions regarding ANC. Previously, Ghanaian women from less affluent families, with lower levels of education, and those who were younger had limited access to ANC even before the pandemic [10]. It is critical to assess how the COVID-19 pandemic interacted with these social determinants of health and other underpinning factors that influence ANC attendance.

The proportions of births attended by trained healthcare workers may have reduced due to possible decline in ANC attendance as a result of COVID-19 thus, resulting in poor maternal health outcomes in low- and middle-income countries (LMICs) [11–13]. Globally, the impact of COVID-19 on maternal and child health has been a major concern. Studies had predicted cutbacks in the number of services covered by overburdened health systems, restrictions on movement, and decreases in care-seeking [14, 15]. It is estimated that a 10% reduction in the availability of basic prenatal and neonatal care resulted in 28,000 and 168,000 more maternal and infant deaths respectively worldwide [16]. An important strategy to reduce maternal mortality in LMICs is increasing the proportion of births attended by skilled personnel [17]. In Ethiopia, this was achieved primarily by increasing the proportion of births delivered in health facilities that offered obstetric care [14]. A study of pregnant women in Nepal found a 52% decline in health facility births pre- and post-COVID-19 lockdown [18]. A follow-up review which compared childbirth during the pandemic found that health facility delivery rate decreased by 45% and 38% during the first and second waves respectively in Nepal [19]. In sub-Saharan Africa (SSA), a significant constraint in determining the impact of COVID-19 on overall maternal health care access was that most studies only included women who successfully obtained ANC services [14].

Globally, preterm births (10.6%) and low birth weight (LBW) (14.6% of all births) are on the decline [20, 21]. However, the COVID-19 pandemic may have negatively impacted fetal growth and raised the incidence of preterm birth and LBW. Previous pandemics (specifically the "Spanish flu") had negative consequences on fetal development resulting in preterm birth and LBW [22–24]. Thus, the infection affected the pregnancy, intrauterine development, and child birthing process [24–26]. Accordingly, maternal morbidity and premature birth were positively correlated with COVID 19 infections during pregnancy [26]. Additionally, COVID-19 resulted in significantly higher anxiety, stress, and depressive symptoms in pregnant women [27, 28]. Consequently, the elevated stress may have a negative impact on the course of the pregnancy, leading to preterm birth, and LBW. However, other studies reported that, the rate of severely and moderately preterm babies reduced in the Netherlands, Ireland and Israel during COVID 19 lockdowns [25, 29, 30]. The prevalence of LBW also reduced during the initial COVID-19 restrictions period [31]. In some studies, there were no reported correlation between lockdown periods and adverse perinatal outcomes [20, 32].

A few studies have measured the impact of COVID-19 on the usage of maternal health services in Ghana [33–35]. However, there is limited information on pregnancy outcomes before and during COVID-19 pandemic in the Tamale Metropolis of northern Ghana. Providing data in this regard may help with policies for maternal care in future pandemics. Therefore, this study assessed pregnancy outcomes before and during COVID-19 pandemic in Tamale Metropolis, Ghana.

## Materials and methods

### Study design and setting

A retrospective cohort study was adopted as described in a previous study [36]. Data were collected from respondents in the Tamale Metropolis of Northern Region, Ghana. Women who had given birth before or during COVID-19 were included in the study. The maternal and child health record books for ANC services were also used as another source of data. Data collection took one month to complete (21^st^ February, 2021 to 21^st^ March 2021).

The population of Tamale Metropolis as reported in the 2021 population and housing census was 374,744; with 185,051 males and 189,693 females. The Metropolis has a total landmass of 646.901.80 sqkm [37].

### Sample size determination and sampling techniques

The sample size for the study was determined by using Fisher’s et al (1998) formula:

$$\text{n}=\frac{{\text{Z}}^{2}\text{P}(1-\text{P})}{{\text{e}}^{2}}$$

Where, n = sample size

Z = the value for corresponding confidence level (that is 1.96 for a 95% confidence level)

P = the estimated value for the proportion of the target population that have the condition of interest is 50%

e = the level of statistical significance set which is 5% with a 95% confidence level.


$$\begin{array}{ll} \text{n} & =\frac{1.96^{2}\;\text{x}\;0.50(1-0.50)}{0.05^{2}}\\ & =\frac{3.84\;\text{x}\;0.50\;\text{x}\;0.05}{0.0025}\\ & =384 \end{array}$$

The sample size for this study was 384 respondents. About 20% of non-responses was added to the sample size making a total of 461. A higher proportion was added because the researchers anticipated low response rate due to the COVID-19 pandemic.

The study employed different techniques to recruit respondents. Multistage sampling technique was adopted in selecting the study communities. The Tamale Metropolis was categorized into three clusters; Tamale North, South and Central. One community was randomly chosen under each cluster. The names of the communities in each cluster were written on pieces of paper, folded and placed inside a box. The box was shaken and the papers thoroughly mixed. One community was picked from the list of communities under each cluster. This method was repeated for all the three clusters to have Kalpohin for north, Sakasaka for central and Vittin for south.

Using ratio and proportion method (probability proportional to size sampling), the number of respondents to be recruited from each cluster was determined. This resulted in the following quotas; 128 for Kalpohin, 141 for Sakasaka and 192 for Vittin. In each of the communities of a cluster, random technique was applied. This technique was achieved by identifying the centre of a selected community and spinning a pen. The research team followed the direction of the pen and interviewed respondents at their household levels. Each household had a chance for only one respondent regardless of the number of women who met the inclusion criteria. Where there was more than one woman in a household, a respondent was decided by toss of a coin.

### Study population/Inclusion criteria

The study respondents were women who delivered before or during COVID-19 pandemic. A woman was considered to have delivered before COVID-19 if the delivery occurred from 31^st^ December 2018 to 30^th^ December 2019. While during COVID-19 was taken as women who delivered from 31^st^ December 2019 to 31^st^ March 2021. Women who belonged to both cohorts (delivered before and during COVID-19) were excluded from the study. Additionally, women with underlying condition (s) or have been infected with COVID-19 virus were excluded from the study to control for confounding.

### Data collection tools and procedure

A structured questionnaire was used to collect data from the respondents. Four research assistants were recruited and trained on the purpose, design and tools of the study. They were fluent in the Dagbani language (main local dialect of inhabitants of Tamale Metropolis). The interviews were conducted using face-to-face approach. Respondents chose either the Dagbani language or English language as their preferred medium for the interviews. The questionnaire had two sections; section A deals with socio-demographic characteristics of the respondents and section B asks questions about pregnancy outcomes before and during COVID-19. Close ended questions were used in the questionnaire. Responses were cross-checked with ANC maternal and child health record books to validate the information and minimize recall bias.

### Measurement of study variables

#### Data processing and analysis

The number of respondents recruited were 450 out of the targeted 461 making a response rate of 98%. The data were entered and stored in Statistical Package for Social Sciences (SPSS) version 25. Then, the data were cleaned before analysis. The results were presented as descriptive statistics including percentages and frequencies. Chi-square test and logistic regression were used to assess the associations between the dependent and independent variables at a confidence level of 95% and 5% alpha (α). A p-value <0.05 was considered statistically significant.

#### Operational definitions

- Normal term/full term: Delivery between 37 and 40 weeks of pregnancy (gestation)
- Preterm: Delivery before 37 weeks of pregnancy
- Post term: Delivery after 40 weeks of pregnancy
- Normal birth weight: 2.5 to 3.9kg of birth weight
- Low birth weight: Birth weight of less than 2.5kg
- Complication: Refers to whether there was any undesired or adverse outcome during or after delivery.

#### Ethics considerations

The ethics approval for this study was received from the School of Medical Sciences/Komfo Anokye Teaching Hospital, Committee on Human Research, Publication and Ethics (ID: CHRPE/AP/494/20). An introductory letter was also received from the UDS, Tamale, Ghana. Additionally, information was anonymously collected to protect respondent’s confidentiality. The respondents were informed of the possible benefits of the study, risk and discomfort involved. Furthermore, respondents were informed that their participation in the research was voluntary, and they could withdraw from the study at any time without consequences. A written informed consent was obtained from each respondent before participating in the study. The research assistants and respondents also adhered to the COVID-19 safety protocols.

## Result

### Socio-demographic characteristics of respondents

A total of 450 questionnaires (98% response rate) were included in the final analysis. About half (51.8% and 52.2%) of the respondents were between 26 and 35 years of age and had no formal education respectively. Over three-quarters (82.2%) of the respondents were from the Dagomba ethnic group with most (94.7%) of them being married. Similarly, most (90.2%) respondents practiced Islam. Additionally, about three out of five (60.4%) respondents described their place of residence as urban and 60.2% of the women lived in households with 1 to 5 persons. Averagely, half (52.0%) of the respondents reported of being unemployed (Table 1).

**Table 1:** Socio-demographic characteristics of respondents who delivered before and during COVID-19.

Variable	Total (n = 450)	Before COVID-19 (n = 142)	During COVID-19 (n = 308)	
	Frequency (%)	Frequency (%)	Frequency (%)	Statistical test
**Age (years)**				p = **0.043**
≤25	164 (36.4)	41 (28.9)	123 (39.9)	
26–35	233 (51.8)	79 (55.6)	154 (50.0)	
≥36	53 (11.8)	22 (15.5)	31 (10.1)	
**Level of education**				p = 0.548
No education	235 (52.2)	78 (54.9)	157 (51.0)	
Primary	48 (10.7)	19 (13.4)	29 (9.4)	
Junior High School	85 (18.9)	23 (16.2)	62 (20.1)	
Senior High School	59 (13.1)	15 (10.6)	44 (14.3)	
Tertiary	23 (5.1)	7 (4.9)	16 (5.2)	
**Marital status**				p = 0.366
Single	24 (5.3)	5 (3.5)	19 (6.2)	
Married	426 (94.7)	137 (96.5)	289 (93.8)	
**Ethnicity**				p = 0.276
Dagomba	370 (82.2)	118 (83.1)	252 (81.8)	
Mamprusi	34 (7.6)	8 (5.6)	26 (8.4)	
Gonja	35 (7.8)	10 (7.0)	25 (8.1)	
Others (e.g. Akan, Frafra, Grusi)	11 (2.4)	6 (4.2)	5 (1.6)	
**Occupation**				p = 0.234
Farmer	30 (6.7)	8 (5.6)	22 (7.1)	
Trader	132 (29.3)	52 (36.6)	80 (26.0)	
Unemployed	234 (52.0)	64 (45.1)	170 (55.2)	
Public servant	21 (4.7)	6 (4.2)	15 (4.9)	
Seamstress	17 (3.7)	7 (4.9)	10 (3.2)	
Others (e.g. Hairdresser, designer)	16 (3.6)	5 (3.5)	11 (3.6)	
**Religion**				p = 0.865
Christianity	44 (9.8)	13 (9.2)	31 (10.1)	
Islam	406 (90.2)	129 (90.8)	277 (89.9)	
**Area of residence**				p = 0.904
Urban	272 (60.4)	88 (62.0)	184 (59.7)	
Peri-urban	76 (16.9)	23 (16.2)	53 (17.2)	
Rural	102 (22.7)	31 (21.8)	71 (23.1)	
**Household size**				p = 0.206
1–5	271 (60.2)	77 (54.2)	194 (63.0)	
6–10	153 (34.0)	56 (39.4)	97 (31.5)	
11 and above	26 (5.8)	9 (6.3)	17 (5.5)	

### Pregnancy outcomes before and during COVID-19

In all, 75.6% of the respondents attended at least one ANC visit. However, the proportion of respondents who attended at least one ANC visit before COVID-19 (86.6%) was higher compared to 70.5% during the pandemic. Also, a high proportion (76.8%) of mothers delivered at a health facility before the pandemic compared to 58.1% during COVID-19. The proportion of preterm births increased from 18.4% to 19.4% before and during COVID-19 respectively. The study also found that the proportion of LBW increased from 17.0% to 20.7% before and during COVID-19 respectively. Similarly, 83.6% of respondents experienced complications during recent delivery. The proportion of reported complications during delivery increased from 78.2% to 86.0% before and during COVID-19 respectively (Table 2).

**Table 2:** Pregnancy outcome before and during COVID-19 in Tamale.

	Total (n = 450)	COVID-19		Statistical test
		Before (n = 142)	During (n = 308)	
	Frequency (%	Frequency (%)	Frequency (%)	
**ANC attendance**				p**<0.001**
Yes	340 (75.6)	123 (86.6)	217 (70.5)	
No	110 (24.4)	19 (13.4)	91 (29.5)	
**Place of delivery**				p**<0.001**
Hospital	288 (64.0)	109 (76.8)	179 (58.1)	
Home	162 (36.0)	33 (23.2)	129 (41.9)	
**Gestational age at delivery**				p = 0.718
Normal term	199 (68.9)	66 (67.3)	133 (69.3)	
Preterm	55 (19.0)	18 (18.4)	37 (19.4)	
Post term	35 (12.1)	14 (14.3)	21 (11.0)	
**Birth weight**				p = 0.718
Normal birth weight	262 (91.0)	101 (92.7)	161 (89.9)	
Low birth weight	26 (9.0)	8 (7.3)	18 (10.1)	
* **Complication during delivery**				**p = 0.041**
No	74 (16.4)	31 (21.8)	43 (14.0)	
Yes	376 (83.6)	111 (78.2)	265 (86.0)	

* Complications included: preterm birth, low birth weight, postpartum bleeding, purpureal infection, maternal distress and prolonged admission

### Socio-demographic characteristics and pregnancy outcomes before and during COVID-19

Bivariate analysis of sociodemographic characteristics and pregnancy outcomes before and during COVID-19 indicates that age was statistically significant in Tamale Metropolis. There was a reduced odds of 0.47 among respondents who were 36 years and above (OR = 0.47, 95%CI: 0.25, 0.90 & p = 0.023) compared to 25 years of age or younger during COVID-19. The respondents ANC visits, place of delivery, and complications during delivery were significantly associated with the COVID-19 period. There was an increased odds of 2.72 of respondents attending no ANC (OR = 2.72, 95%CI: 1.58, 1.67 & p<0.001) during COVID-19 compared to one or more attendance. The study also found that there was 2.38 increased odds of home deliveries among the respondents (OR = 2.38, 95%CI: 1.52, 3.74 & p<0.001) during COVID-19. Similarly, the odds of respondents experiencing complications during COVID-19 also increased (OR = 1.72, (95%CI: 1.03, 2.87 & p = 0.041) (Table 3).

**Table 3:** Socio-demographic characteristics and pregnancy outcome before and during COVID-19.

	COVID-19			
	Before (n = 142)	During (n = 308)		
	Freq (%)	Freq (%)	OR (95%CI)	p-value
**Age (years)**				
≤25	41 (28.9)	123 (39.9)	Ref	
26–35	79 (55.6)	154 (50.0)	0.65 (0.42–1.02)	0.058
≥36	22 (15.5)	31 (10.1)	0.47 (0.25–0.90)	**0.023**
**Level of education**				
No education	78 (54.9)	157 (51.0)	Ref	
Primary	19 (13.4)	29 (9.4)	0.76 (0.40–1.44)	0.396
Junior High School	23 (16.2)	62 (20.1)	1.34 (0.77–2.32)	0.298
Senior High School	15 (10.6)	44 (14.3)	1.46 (0.76–2.78)	0.253
Tertiary	7 (4.9)	16 (5.2)	1.14 (0.45–2.88)	0.788
**Marital status**				
Single	5 (3.5)	19 (6.2)	Ref	
Married	137 (96.5)	289 (93.8)	0.56 (0.20–1.52)	0.251
**Ethnicity**				
Dagomba	118 (83.1)	252 (81.8)	Ref	
Mamprusi	8 (5.6)	26 (8.4)	1.52 (0.67–3.46)	0.317
Gonja	10 (7.0)	25 (8.1)	1.17 (0.55–2.52)	0.687
Others (e.g. Akan, Frafra, Grusi)	6 (4.2)	5 (1.6)	0.39 (0.12–1.30)	0.126
**Occupation**				
Farmer	8 (5.6)	22 (7.1)	Ref	
Trader	52 (36.6)	80 (26.0)	0.56 (0.23–1.35)	0.196
Unemployed	64 (45.1)	170 (55.2)	0.97 (0.41–2.28)	0.937
Public servant	6 (4.2)	15 (4.9)	0.91 (0.26–3.16)	0.881
Seamstress	7 (4.9)	10 (3.2)	0.80 (0.21–3.03)	0.743
Others (e.g. Hairdresser, designer)	5 (3.5)	11 (3.6)	0.52 (0.15–1.83)	0.308
**Religion**				
Christianity	13 (9.2)	31 (10.1)	Ref	
Islam	129 (90.8)	277 (89.9)	0.90 (0.46–1.78)	0.763
**Area of residence**				
Urban	88 (62.0)	184 (59.7)	Ref	
Peri-urban	23 (16.2)	53 (17.2)	1.10 (0.64–1.91)	0.730
Rural	31 (21.8)	71 (23.1)	1.10 (0.67–1.79)	0.717
**Household size**				
1–5	77 (54.2)	194 (63.0)	Ref	
6–10	56 (39.4)	97 (31.5)	0.67 (0.45–1.05)	0.082
11 and above	9 (6.3)	17 (5.5)	0.75 (0.32–1.75)	0.507
**ANC attendance**				
Yes	123 (86.6)	217 (70.5)	Ref	
No	19 (13.4)	91 (29.5)	2.72 (1.58–1.67)	**<0.001**
**Place of delivery**				
Hospital	109 (76.8)	179 (58.1)	Ref	
Home	33 (23.2)	129 (41.9)	2.38 (1.52–3.74)	**<0.001**
**Gestational age at delivery**				
Normal term	66 (67.3)	133 (69.3)	Ref	
Preterm	18 (18.4)	37 (19.4)	1.02 (0.54–1.93)	0.951
Post term	14 (14.3)	21 (11.0)	0.74 (0.356–1.56)	0.433
**Birth weight**				
Normal birth weight	39 (83.0)	69 (79.3)	Ref	
Low birth weight	8 (17.0)	18 (20.7)	3.12 (0.88–11.04)	0.437
* **Any complication (s) during delivery**				
No	31 (21.8)	43 (14.0)	Ref	
Yes	111 (78.2)	265 (86.0)	1.72 (1.03–2.87)	**0.041**

* Complications included: preterm birth, low birth weight, postpartum bleeding, purpureal infection, maternal distress and prolonged admission

## Discussion

This study assessed pregnancy outcomes before and during the COVID-19 pandemic in Tamale Metropolis, Ghana. Socio-demographic characteristics, antenatal care (ANC) attendance, place of delivery, complications during delivery (including preterm, low birth weight, postpartum bleeding, purpureal infection, and prolonged admission) were measured in the study.

The ANC attendance during the pandemic reduced from 86.6% to 70.5%. Similarly, women who delivered during the pandemic were 2.8 times more likely not to attend ANC than women who delivered before COVID-19. This finding is similar to previous studies which reported that lockdown and movement restrictions affected ANC attendance during the COVID-19 pandemic in Ghana Ethiopia [38, 39]. These studies further reported that pregnant women were unable to access health care compared to the period before the COVID-19 pandemic. A review of studies confirmed that more pregnant women were attending ANC before COVID-19 compared to the lockdown period [40]. The study reported that COVID-19 pandemic could have negatively influenced the ANC attendance among pregnant women [40]. However, studies from eight sub-Saharan African countries reported that pregnant women continued to utilize ANC services during the COVID-19 pandemic [34]. The difference could be due to the effect of COVID-19 in the study setting. In context with high incidence of COVID-19 also experienced strict restrictions. Consequently, disruptions in the daily routines of people affected access to basic health care and utilization of maternal health services. Some factors such as distance to health facility, level of maternal education, and age have been described as enablers of pregnant women accessing and utilizing ANC services during COVID-19 pandemic [34].

Our study also found that the proportion of pregnant women who delivered at home during the COVID-19 pandemic was 2.38 times more compared to before. This finding is similar to previous studies which reported that COVID-19 influenced choice of delivery place of pregnant women [41, 42]. Before the COVID-19 pandemic, pregnant women made the choice to deliver at places they considered safe. Likewise, women considered the home to be safer to deliver their babies compared to the health facilities during the COVID-19 pandemic. This resulted in low health facility deliveries and low skilled births. In France, some pregnant women held the view that health facility delivery was unsafe compared to home delivery due to the fear of spread of the Corona virus [43]. Additionally, there was a directive by the Government of Ghana that pregnant women should not go out of their homes including utilization of the health care unless it was critical [6]. This directive coupled with lack of education on birth preparedness and complication readiness predisposed expectant mothers to home deliveries.

In our study, preterm delivery increased slightly from 18.4% before the pandemic to 19.4% during COVID-19 but the difference is not statistically significant. This finding is similar to a Turkish study which reported that preterm births significantly increased during the pandemic [26]. However, another study conducted in Netherlands reported that preterm births rather decreased during COVID-19 pandemic [25]. The varied findings may be due to geographical differences in the study settings. The adverse impact of the COVID-19 and experiences of countries varied [44]. Our study showed that increased preterm births could be due to negative experiences and impact of the COVID-19 pandemic in Northern Region of Ghana. The pandemic might have prevented pregnant women from accessing quality health care and consequently resulting in adverse birth outcomes such as preterm deliveries.

Our study also found that the proportion of low birth weight increased from 17.0% to 20.7% before and during COVID-19 respectively but the difference is not statistically significant. This finding is similar to a study that compared birth weights before and during COVID-19 pandemic. It was reported that more LBW neonates were delivered during lockdown [45]. Other studies maintained that the lockdown restrictions had no adverse effects on birth weight [31, 32].

In this study, women who delivered during COVID-19 were twice more likely to experience complications compared to those who had their babies before COVID-19. This finding is similar to that of a study conducted by Cousins (2020) to assess the link between COVID-19 and adverse birth outcomes. A systematic review reported that pregnant women experienced complications during delivery which was comprehensively linked to the COVID-19 pandemic [46]. The findings showed that majority of the pregnant women experienced prolonged labor and postpartum hemorrhage. Other studies also suggested that COVID-19 was associated with an increased risk of adverse birth outcomes, including iatrogenic preterm birth and increased rates of cesarean section delivery compared to the period before the pandemic [47–49]. This complication maybe as a result of inability of the women to attendant ANC regularly due to COVID-19 restrictions. The likelihood of adverse birth outcomes among pregnant women with little information of ANC will be higher when compared to knowledgeable women [50].

### Strengths and limitations

This study has strengths. A multistage sampling approach ensured all communities in the Tamale Metropolis had equal chances of being picked. Therefore, the findings reflect a representative sample of the study population. Simple random sampling method was applied to mitigate bias in respondents’ selection. Additionally, the study is community based and respondents answered questions within familiar settings. We also had access to the maternal and child health record books for review and information extraction. However, this study also has some limitations. Reliability of information was based on respondents’ ability to remember correctly, hence, findings are liable to recall bias. The sample size for the before and during COVID-19 varied, as well as difference in their ages could limit generalization of the findings The study is also liable to selection bias due to exclusion of women with comorbidities (including COVID-19), women who died, and women who were pregnant more than once during the study period. This study is also limited geographically and the findings should be interpreted with care when applied in different contexts.

## Conclusion

In this study, the ANC attendance and health facility delivery decreased during the pandemic. During disease outbreaks, outreach engagement strategies should be developed and implemented to increase access and utilization of maternal health services for marginalized and underserved populations. The capacity of health workers should be strengthened through skill training to appropriately manage adverse birth outcomes.

## Supporting information

S1 Data set (XLSX)

## References

[1] Huang C, Wang Y, Li X, Ren L, Zhao J, Hu Y, et al. Clinical features of patients infected with 2019 novel coronavirus in Wuhan, China. The lancet. 2020;395(10223):497–506. 10.1016/S0140-6736(20)30183-5 PMC7159299 31986264

[2] Wang C, Horby PW, Hayden FG, Gao GF. A novel coronavirus outbreak of global health concern. The lancet. 2020;395(10223):470–3. doi: 10.1016/S0140-6736(20)30185-9 31986257 PMC7135038

[3] World Health Organization. Coronavirus disease (COVID-2019) situation reports [Internet]. 2020 [cited 10 April 2021]. https://reliefweb.int/report/world/coronavirus-disease-2019-covid-19-situation-report-94-23-april-2020?gad_source=1&gclid=Cj0KCQjw2PSvBhDjARIsAKc2cgMbOPNglG68wYvLbxoYqdTOhdgjePVm1GNJGhtMWNJ5QdskWctv_SIaAgYdEALw_wcB

[4] Ghana’s Outbreak Response Management Updates [Internet]. 2020 [cited 10 September 2021]. https://www.ghanahealthservice.org/covid19/.

[5] Morhe EKS, Anto EO, Coall DA, Adua E, Debrah AY, Addai-Mensah O, et al. SARS-CoV-2 updates in a West African population and precautionary measures for sustaining quality antenatal care delivery. Journal of Global Health. 2020;10(2). 10.7189/jogh.10.020365 PMC7567409 33110560

[6] Sarkodie B, Asiedu-Bekoe F, Laryea DO, Ampofo WK, Phillips RO, Samba A, et al. Overview of preparedness and response to COVID-19 in Ghana. Ghana medical journal. 2021;55(2):38–47. doi: 10.4314/gmj.v55i2s.6 35233113 PMC8853692

[7] World Health Organization. Pulse survey on continuity of essential health services during the COVID-19 pandemic: interim report, 27 August 2020. World Health Organization; 2020.

[8] Asuming PO, Gaisie DA, Agula C, Bawah AA. Impact of Covid-19 on Maternal Health Seeking in Ghana. Journal of international development. 2022;34(4):919–30. doi: 10.1002/jid.3627 35465457 PMC9015309

[9] Yerger P, Jalloh M, Coltart CE, King C. Barriers to maternal health services during the Ebola outbreak in three West African countries: a literature review. BMJ global health. 2020;5(9):e002974. doi: 10.1136/bmjgh-2020-002974 32895217 PMC7476472

[10] Gudu W, Addo B. Factors associated with utilization of skilled service delivery among women in rural Northern Ghana: a cross sectional study. BMC Pregnancy Childbirth. 2017;17:1–10. 28566088 10.1186/s12884-017-1344-2 PMC5452376

[11] Chou VB, Walker N, Kanyangarara M. Estimating the global impact of poor quality of care on maternal and neonatal outcomes in 81 low-and middle-income countries: a modeling study. PLoS medicine. 2019;16(12):e1002990. doi: 10.1371/journal.pmed.1002990 31851685 PMC6919595

[12] Metwally AM, Abdel-Latif GA, Mohsen A, El Etreby L, Elmosalami DM, Saleh RM, et al. Strengths of community and health facilities based interventions in improving women and adolescents’ care seeking behaviors as approaches for reducing maternal mortality and improving birth outcome among low income communities of Egypt. BMC Health Services Research. 2020;20:1–14. doi: 10.1186/s12913-020-05412-1 32600377 PMC7322855

[13] Karkee R, Tumbahanghe KM, Morgan A, Maharjan N, Budhathoki B, Manandhar DS. Policies and actions to reduce maternal mortality in Nepal: perspectives of key informants. Sexual and Reproductive Health Matters. 2022;29(2):1907026. 10.1080/26410397.2021.1907026 PMC8032335 33821780

[14] Zimmerman LA, Desta S, Karp C, Yihdego M, Seme A, Shiferaw S, et al. Effect of the COVID-19 pandemic on health facility delivery in Ethiopia; results from PMA Ethiopia’s longitudinal panel. PLOS Global Public Health. 2021;1(10):e0000023. doi: 10.1371/journal.pgph.0000023 36962067 PMC10021675

[15] Akseer N, Kandru G, Keats EC, Bhutta ZA. COVID-19 pandemic and mitigation strategies: implications for maternal and child health and nutrition. The American journal of clinical nutrition. 2020;112(2):251–6. doi: 10.1093/ajcn/nqaa171 32559276 PMC7337702

[16] Meherali S, Adewale B, Ali S, Kennedy M, Salami B, Richter S, et al. Impact of the COVID-19 pandemic on adolescents’ sexual and reproductive health in low-and middle-income countries. International Journal of Environmental Research and Public Health. 2021;18(24):13221. doi: 10.3390/ijerph182413221 34948829 PMC8701118

[17] Nelson JR, Ess RH, Dickerson TT, Gren LH, Benson LS, Manortey SO, et al. Strategies to increase rural maternal utilization of skilled health personnel for childbirth delivery in low-and middle-income countries: a narrative review. Global Health Action. 2022;15(1):2058170. doi: 10.1080/16549716.2022.2058170 35506937 PMC9090426

[18] Ashish K, Gurung R, Kinney MV, Sunny AK, Moinuddin M, Basnet O, et al. Effect of the COVID-19 pandemic response on intrapartum care, stillbirth, and neonatal mortality outcomes in Nepal: a prospective observational study. The lancet Global health. 2020;8(10):e1273–e81. doi: 10.1016/S2214-109X(20)30345-4 32791117 PMC7417164

[19] Meena D, Puri M, Jaiswal N, Kumar M, Handa A, Nain S. Impact of different waves of COVID-19 on the rate and indications of Caesarean delivery: An Observational Study. Nepal Journal of Obstetrics and Gynaecology. 2022;17(34):85–91.

[20] Vaccaro C, Mahmoud F, Aboulatta L, Aloud B, Eltonsy S. The impact of COVID-19 first wave national lockdowns on perinatal outcomes: a rapid review and meta-analysis. BMC Pregnancy Childbirth. 2021;21:1–14. 34615505 10.1186/s12884-021-04156-y PMC8532086

[21] Qian T, Zhang R, Zhu L, Shi P, Yang J, Yang C-y, et al. Necrotizing enterocolitis in low birth weight infants in China: mortality risk factors expressed by birth weight categories. Pediatrics & Neonatology. 2017;58(6):509–15. 28528756 10.1016/j.pedneo.2016.10.004

[22] He J, Liu Z-W, Lu Y-P, Li T-Y, Liang X-J, Arck PC, et al. A systematic review and meta-analysis of influenza a virus infection during pregnancy associated with an increased risk for stillbirth and low birth weight. Kidney and Blood Pressure Research. 2017;42(2):232–43. doi: 10.1159/000477221 28514782

[23] Eaton A, Lewis N, Fireman B, Hansen J, Baxter R, Gee J, et al. Birth outcomes following immunization of pregnant women with pandemic H1N1 influenza vaccine 2009–2010. Vaccine. 2018;36(19):2733–9. doi: 10.1016/j.vaccine.2017.08.080 28917536 PMC6708558

[24] Papapanou M, Papaioannou M, Petta A, Routsi E, Farmaki M, Vlahos N, et al. Maternal and neonatal characteristics and outcomes of COVID-19 in pregnancy: an overview of systematic reviews. International journal of environmental research and public health. 2021;18(2):596. doi: 10.3390/ijerph18020596 33445657 PMC7828126

[25] Been JV, Ochoa LB, Bertens LC, Schoenmakers S, Steegers EA, Reiss IK. Impact of COVID-19 mitigation measures on the incidence of preterm birth: a national quasi-experimental study. The Lancet Public Health. 2020;5(11):e604–e11. doi: 10.1016/S2468-2667(20)30223-1 33065022 PMC7553867

[26] Sentilhes L, De Marcillac F, Jouffrieau C, Kuhn P, Thuet V, Hansmann Y, et al. Coronavirus disease 2019 in pregnancy was associated with maternal morbidity and preterm birth. American journal of obstetrics and gynecology. 2020;223(6):914.e1–.e15. doi: 10.1016/j.ajog.2020.06.022 32553908 PMC7294260

[27] Ayaz R, Hocaoğlu M, Günay T, devrim Yardımcı O, Turgut A, Karateke A. Anxiety and depression symptoms in the same pregnant women before and during the COVID-19 pandemic. Journal of perinatal medicine. 2020;48(9):965–70. doi: 10.1515/jpm-2020-0380 32887191

[28] Stepowicz A, Wencka B, Bieńkiewicz J, Horzelski W, Grzesiak M. Stress and anxiety levels in pregnant and post-partum women during the COVID-19 pandemic. International journal of environmental research and public health. 2020;17(24):9450. doi: 10.3390/ijerph17249450 33348568 PMC7766953

[29] Meyer R, Bart Y, Tsur A, Yinon Y, Friedrich L, Maixner N, et al. A marked decrease in preterm deliveries during the coronavirus disease 2019 pandemic. American Journal of Obstetrics & Gynecology. 2021;224(2):234–7. doi: 10.1016/j.ajog.2020.10.017 33069683 PMC7560113

[30] McDonnell S, McNamee E, Lindow SW, O’Connell MP. The impact of the Covid-19 pandemic on maternity services: A review of maternal and neonatal outcomes before, during and after the pandemic. European Journal of Obstetrics & Gynecology and Reproductive Biology. 2020;255:172–6. doi: 10.1016/j.ejogrb.2020.10.023 33142263 PMC7550066

[31] Kirchengast S, Hartmann B. Pregnancy outcome during the first COVID 19 lockdown in Vienna, Austria. International journal of environmental research and public health. 2021;18(7):3782. doi: 10.3390/ijerph18073782 33916365 PMC8038559

[32] Caniglia EC, Magosi LE, Zash R, Diseko M, Mayondi G, Mabuta J, et al. Modest reduction in adverse birth outcomes following the COVID-19 lockdown. American journal of obstetrics and gynecology. 2021;224(6):615.e1–.e12. doi: 10.1016/j.ajog.2020.12.1198 33347842 PMC7817370

[33] Ahmed T, Rahman AE, Amole TG, Galadanci H, Matjila M, Soma-Pillay P, et al. The effect of COVID-19 on maternal newborn and child health (MNCH) services in Bangladesh, Nigeria and South Africa: call for a contextualised pandemic response in LMICs. International Journal for Equity in Health. 2021;20(1):1–6. 33722225 10.1186/s12939-021-01414-5 PMC7957460

[34] Tadesse E. Antenatal care service utilization of pregnant women attending antenatal care in public hospitals during the COVID-19 pandemic period. International journal of women’s health. 2020;12:1181–1188. doi: 10.2147/IJWH.S287534 33335430 PMC7737544

[35] Shapira G, Ahmed T, Drouard SHP, Amor Fernandez P, Kandpal E, Nzelu C, et al. Disruptions in maternal and child health service utilization during COVID-19: analysis from eight sub-Saharan African countries. Health policy and planning. 2021;36(7):1140–51. doi: 10.1093/heapol/czab064 34146394 PMC8344431

[36] Molina RL, Tsai TC, Dai D, Soto M, Rosenthal N, Orav EJ, et al. Comparison of pregnancy and birth outcomes before vs during the COVID-19 pandemic. JAMA Network Open. 2022;5(8):e2226531–e. doi: 10.1001/jamanetworkopen.2022.26531 35960517 PMC9375166

[37] Ghana Statistical Service. Population and Housing Census: Population of Regions and Districts. Ghana Statistical Service. 2021.

[38] UNFPA’S COVID-19 Response in Ghana [Internet]. 2020 [cited 15 June 2021]. https://ghana.unfpa.org/en/publications/covid-chronicles-unfpas-covid-19-response-ghana.

[39] Norris KG, Huang PA, Glantz JC, Kodam R-S, Anto-Ocrah M. A Cross-Cultural Analysis of the COVID-19 Pandemic’s Impact on Antenatal Healthcare-Seeking Behaviors in Ghana and the United States. Journal of Patient Experience. 2021;8:23743735211062392. doi: 10.1177/23743735211062392 34869849 PMC8640296

[40] Karpati J, Elezaj E, Cebotari V, de Neubourg C. Primary and secondary impacts of the COVID-19 pandemic on children in Ghana. 2021.

[41] Mullins E, Evans D, Viner R, O’Brien P, Morris E. Coronavirus in pregnancy and delivery: rapid review. Ultrasound in Obstetrics & Gynecology. 2020;55(5):586–92. doi: 10.1002/uog.22014 32180292

[42] Ameyaw EK, Ahinkorah BO, Seidu A-A, Njue C. Impact of COVID-19 on maternal healthcare in Africa and the way forward. Archives of Public Health. 2021;79(1):1–5. 34886893 10.1186/s13690-021-00746-6 PMC8660651

[43] Nosratabadi M, Sarabi N, Masoudiyekta L. A case report of vaginal delivery at home due to fear of Covid-19. Iranian journal of psychiatry. 2020;15(4):366. doi: 10.18502/ijps.v15i4.4306 33240387 PMC7610078

[44] Caki N, Krupić D, Corr P. Pyschosocial effects of the COVID-19 pandemic. The societal impacts of COVID-19: A transnational perspective. 2021:63–78.

[45] Garabedian C, Dupuis N, Vayssière C, Bussières L, Ville Y, Renaudin B, et al. Impact of COVID-19 lockdown on preterm births, low birthweights and stillbirths: A retrospective cohort study. Journal of Clinical Medicine. 2021;10(23):5649. doi: 10.3390/jcm10235649 34884351 PMC8658711

[46] Cousins S. COVID-19 has “devastating” effect on women and girls. The Lancet. 2020;396(10247):301–2. doi: 10.1016/S0140-6736(20)31679-2 32738942 PMC7392550

[47] Yang R, Mei H, Zheng T, Fu Q, Zhang Y, Buka S, et al. Pregnant women with COVID-19 and risk of adverse birth outcomes and maternal-fetal vertical transmission: a population-based cohort study in Wuhan, China. BMC medicine. 2020;18(1):1–7. 33070775 10.1186/s12916-020-01798-1 PMC7568966

[48] Chmielewska B, Barratt I, Townsend R, Kalafat E, van der Meulen J, Gurol-Urganci I, et al. Effects of the COVID-19 pandemic on maternal and perinatal outcomes: a systematic review and meta-analysis. The Lancet Global Health. 2021;9(6):e759–e72. doi: 10.1016/S2214-109X(21)00079-6 33811827 PMC8012052

[49] Ranjbar F, Allahqoli L, Ahmadi S, Mousavi R, Gharacheh M, Eshraghi N, et al. Changes in pregnancy outcomes during the COVID-19 lockdown in Iran. BMC Pregnancy and Childbirth. 2021;21(1):1–6. 34420514 10.1186/s12884-021-04050-7 PMC8380188

[50] Allotey J, Stallings E, Bonet M, Yap M, Chatterjee S, Kew T, et al. Clinical manifestations, risk factors, and maternal and perinatal outcomes of coronavirus disease 2019 in pregnancy: living systematic review and meta-analysis. Bmj. 2020;370. doi: 10.1136/bmj.m3320 32873575 PMC7459193

